# Rare Germline Variants in DNA Repair Genes Detected in *BRCA*-Negative Finnish Patients with Early-Onset Breast Cancer

**DOI:** 10.3390/cancers16172955

**Published:** 2024-08-24

**Authors:** Viivi Kurkilahti, Venkat Subramaniam Rathinakannan, Erja Nynäs, Neha Goel, Kristiina Aittomäki, Heli Nevanlinna, Vidal Fey, Minna Kankuri-Tammilehto, Johanna Schleutker

**Affiliations:** 1 Cancer Research Unit and FICAN West Cancer Centre, Institute of Biomedicine, University of Turku and Turku University Hospital, 20014 Turku, Finland; vilolai@utu.fi (V.K.);; 2 Department of Obstetrics and Gynecology, University of Helsinki and Helsinki University Hospital, 00280 Helsinki, Finland; 3 Department of Clinical Genetics, University of Helsinki and Helsinki University Hospital, 00250 Helsinki, Finland; 4 Faculty of Medicine and Health Technology/BioMediTech, Tampere University, 33520 Tampere, Finland; 5 Department of Clinical Genetics, Turku University Hospital, 20520 Turku, Finland; mikanku@utu.fi; 6 Department of Genomics, Laboratory Division, Turku University Hospital, 20520 Turku, Finland

**Keywords:** Breast cancer, early onset, BRCA1/2 negative, DNA repair genes, rare variants

## Abstract

**Simple Summary:**

Breast cancer is the most common cancer in females. Although rare in the younger population, individuals with a susceptible genetic background are at higher risk of breast cancer. A study was conducted on 63 Finnish breast cancer patients without any *BRCA1/2* variants who had an onset of breast cancer at age 40 or younger. These patients were sequenced, and variants in DNA repair genes were identified. These variants were then prioritized based on their allele frequency in the population and pathogenicity prediction scores to identify potential new risk variants. Seventy-two deleterious variants were found, including eight novel variants. For the novel variants, protein structure modeling was conducted, and all deleterious variants were validated in another Finnish *BRCA1/2*-negative breast cancer population.

**Abstract:**

Background: Breast cancer is the most common malignancy, with a mean age of onset of approximately 60 years. Only a minority of breast cancer patients present with an early onset at or before 40 years of age. An exceptionally young age at diagnosis hints at a possible genetic etiology. Currently, known pathogenic genetic variants only partially explain the disease burden of younger patients. Thus, new knowledge is warranted regarding additional risk variants. In this study, we analyzed DNA repair genes to identify additional variants to shed light on the etiology of early-onset breast cancer. Methods: Germline whole-exome sequencing was conducted in a cohort of 63 patients diagnosed with breast cancer at or before 40 years of age (median 33, mean 33.02, range 23–40 years) with no known pathogenic variants in *BRCA* genes. After filtering, all detected rare variants were sorted by pathogenicity prediction scores (CADD score and REVEL) to identify the most damaging genetic changes. The remaining variants were then validated by comparison to a validation cohort of 121 breast cancer patients with no preselected age at cancer diagnosis (mean 51.4 years, range 28–80 years). Analysis of novel exonic variants was based on protein structure modeling. Results: Five novel, deleterious variants in the genes *WRN*, *RNF8*, *TOP3A*, *ERCC2*, and *TREX2* were found in addition to a splice acceptor variant in *RNF4* and two frameshift variants in *EXO1* and *POLE* genes, respectively. There were also multiple previously reported putative risk variants in other DNA repair genes. Conclusions: Taken together, whole-exome sequencing yielded 72 deleterious variants, including 8 novel variants that may play a pivotal role in the development of early-onset breast cancer. Although more studies are warranted, we demonstrate that young breast cancer patients tend to carry multiple deleterious variants in one or more DNA repair genes.

## 1. Introduction

Breast cancer (BC) is the most common cancer among females worldwide and the second leading cause of cancer deaths in European countries, including Finland [1]. BC is usually diagnosed after menopause, with only 4–6% of BCs diagnosed prior to the age of 40 years [2,3]. Patients with early-onset BC (EOBC) tend to exhibit poorer prognosis likely due to its more aggressive tumor subtypes [4]. Among EOBC patients, *BRCA1/2* mutation carriers tend to have a poorer prognosis [5].

BC is a heterogeneous group of diseases that can be classified into subtypes according to responses to therapies or according to the expression of molecular features [6,7]. EOBC patients have more triple-negative subtype tumors (TNBC) [8]. TNBC comprises the most aggressive cluster of all breast cancer types. TNBCs present with a rapid progression, a high probability of early recurrence and distant metastasis, and account for 15–20% of all BC cases [9]. 

Genetic predisposition is a pivotal risk factor particularly for developing EOBC and also BC at a later age. Up to 10% of all BC cases are estimated to be hereditary with an underlying high breast cancer risk, but only a fraction of BCs is associated with pathogenic variants (PVs) in *BRCA1/2* genes [10]. Approximately 20 years ago in Finland, pathogenic *BRCA1/2* variants were observed in 25% of high-risk breast and ovarian cancer families [11]. This has decreased over time in Finland as well as worldwide due to the refining of referral criteria and their easy discoverability online as well as widened gene test criteria and technological improvements in testing. Currently, in southwestern Finland, the amount of pathogenic *BRCA1/2* variants is approximately 10% in all high-risk breast and ovarian cancer families. However, regional variations in the *BRCA1/2* variants and their frequencies have been observed [12]. In addition to *BRCA1/2*, several other high-risk cancer susceptibility genes have been previously found. In the Finnish population, also unique low and intermediate risk alleles have been identified [13]. The polygenic risk model explains the combined effect of genetic predisposition with additional variants that together create the overall cancer risk [14,15]. Thus, patients with aggressive and EOBC may carry several PVs that together lead to an elevated risk of developing breast cancer at an early age [10]. 

To date, the genetic predisposition factors remain unidentified in many EOBC patients despite increasing knowledge of PVs. To analyze the contribution of rare variants to the development of diseases such as BC, these variants require identification by DNA sequencing. As genome sequencing continues to identify more rare variants, their role in diseases will also become clearer with the accumulating data [16]. Rare variants can have distinctive and unique roles in gene function and expression, and they can display a larger population specificity, which present excellent possibilities for candidates of precision medicine. In this study, whole-exome sequencing (WES) and the created pipeline for variant calling and prioritization were used to identify novel and rare risk variants in DNA repair genes. To elucidate the role of these deleterious variants in the Finnish population, we carefully analyzed the clinical picture, validated the findings in another cohort of Finnish BC patients with a family history of BC and without known BRCA1/2 variants, and created protein modeling for novel variants.

## 2. Methods

### 2.1. Turku Whole-Exome Sequencing Set

#### 2.1.1. Study Subjects

Genomic DNA samples from the blood of 63 individuals were used for WES. Study subjects were females with BC who had received genetic counseling on BC susceptibility in the Turku University Hospital between 1996 and 2018. The Department of Clinical Genetics at the University Hospital District of Southwest Finland provides a high level of specialized health care for three hospital districts. Some patients in our study received their cancer treatments in another hospital district, but the genetic counseling took place in Turku. All patients had previously provided a signed informed consent form. The Ethics Committee of the Hospital District of Southwest Finland approved the study. 

All 63 patients were diagnosed with BC at the age of 40 years or younger (median 33, average 33.02, range 23–40 years). The cut-off of 40 years was selected based on the oncology literature [2,17]. Among the 63 patients, 18 (28.6%) were tested due to their young age of onset without a family history of breast or ovarian cancer, and others fulfilled the modified Lund criteria, which is used in the clinical evaluation of familial BC risk [12]. Seventeen (27%) patients were younger than 30 years at the time of the diagnosis, twelve patients (19%) suffered from TNBC, and five individuals (7.9%) were included in both subgroups. Twelve patients (19%) had bilateral BC (Table 1).

Clinical and histological parameters were obtained from the pathology reports. They included hormonal receptor status, HER2-mutational status, tumor grade, tumor histology, and age at diagnosis. Here, TNBC is defined as a tumor with the absence or very low levels (0–2%) of cells expressing ER and PR and an absence of HER2 overexpression [18]. The family history of cancer was obtained from the pedigrees and medical data used in the genetic counseling. An inclusion criterion was no known PV in prior *BRCA1/2* analyzes. For population controls, we used genomic data from the gnomAD database [19].

#### 2.1.2. Sample Preparation and Whole-Exome Sequencing

For whole-exome sequencing, we used genomic DNA, which was extracted from blood leukocytes with the Cytiva Nucleon DNA Extraction Kit BACC3 (Illustra, Fisher Scientific, Waltham, MA, USA). Exome capture and sequencing were conducted by CeGaT (Tübingen, Germany) with the Illumina HiSeq instrument (Illumina, Inc., San Diego, CA, USA). Library preparation was performed with 1 µg of genomic DNA per sample by the Agilent SureSelectXT Library Prep Kit and Agilent Sure SelectXT Human All Exon V6 enrichment kit (Agilent, Santa Clara, CA, USA). Genome coverage depth was on average 50× per sample.

#### 2.1.3. Data Analysis

The pipeline for the analysis of the data was programmed using Nextflow [20], and each step of the pipeline was implemented as a module. This enabled us to store the results after each step in the analysis process, so that any failure in one of the steps does not require the entire process to be repeated. The quality control for the raw fastq files was performed via FASTQC [21]. The preprocessing step involved removing the adaptor sequences using the tool CutAdapt [22], so that reads below 70 bp and mapping quality lower than 20 were removed. The processed reads were aligned using the BWA-MEM [23] alignment tool against human reference genome (Hg38). Variant calling was conducted using GATK Haplotypecaller [24] and DeepVariant [25]. The resulting vcf files generated from the two variant callers were then combined to give a consensus file, which had the common SNPs and InDels from both callers.

Variant call files from all patients were combined using bcftools [26] to arrive at a single VCF file which was annotated using the Ensembl Variant Effect Predictor (VEP) [27] and downloaded cache files for assembly version GRCh38. For filtering, variants in the merged file were split into SNVs and InDels as the two classes require different approaches.

#### 2.1.4. Variant Filtering

The filtering procedure was performed in R [28] and on the Linux command line, as well as by using biostatistical add-on packages for both platforms.

Both SNV and InDel variants were first tested for missing CADD scores [29]. This step was necessary since, in particular, InDels are not fully scored automatically due to the vast number of possible variations. As expected, missing CADD scores were found only in the InDel files. The following steps were conducted for both SNVs and InDels in the same way.

In the first filtering step, only variants in DNA repair genes were retained. The gene symbols for filtering were obtained from https://www.mdanderson.org/documents/Labs/Wood-Laboratory/human-dna-repair-genes.html [30], where an updated list of DNA repair genes is maintained. The symbols were then updated to their current version and converted to Ensembl Gene IDs by means of functions in the converted R package (https://cran.r-project.org/package=convertid, accessed on 21 March 2022). The resulting DNA repair gene variants were subjected to a general filter excluding variants with a CADD score smaller than 20 and non-canonical transcript variants. In addition to canonical transcript variants, all non-transcript variants were retained as well as all variants with missing CADD scores.

Next, variants were split into two groups for frequency filtering, rare variants with an allele frequency (AF) smaller than 0.01, and ultra-rare variants with no AF reported. AFs were obtained from the gnomAD database [31,32], where both the frequencies calculated from the exome sequencing cohort (gnomADe) and the frequencies calculated from the genome sequencing cohort (gnomADg) had to meet the threshold (or were missing). Exome AFs were obtained from gnomAD version 2 and genome AFs from gnomAD version 3. Both rare and ultra-rare variants were extracted, filtering each by the frequencies calculated for the gnomAD global cohort (gnomADe_AF and gnomADg_AF) and the gnomAD FIN cohort (gnomADe_FIN_AF and gnomADg_FIN_AF).

To arrive at the two main prioritization groups, all variants from the previous step were filtered by “Consequence”. Group 1 has variants that were categorized as “splice_site–altering”, “stop_gain”, “start_lost”, or “non_coding_transcript_exon” variants. Group 2 has only “non-synonymous” (e.g., frameshift) or “missense” variants. Group 2 was further filtered by the REVEL (Rare Exome Variant Ensemble Learner) score [33] for likely pathogenic variants using a threshold of 0.75. Variants with missing REVEL scores were also retained. These variants are considered subcategory 1 (Table 1).

Since the original project objective was to investigate DNA repair genes, the variant locations identified in the previous filtering round were used to obtain a list of genes affected by those variants. To discover any other less harmful variants in the same genes, the filtering was then repeated, starting with all variants found in the original merged VCF that were then mapped to the affected genes. The thresholds for allele frequency and REVEL score were relaxed, using AF < 0.02, which excludes common variants, and REVEL > 0.4, a value chosen according to the comparison of sensitivity and specificity in the supplementary section of the REVEL publication [33]. These variants are considered subcategory 2 (Table 1).

### 2.2. Validation Set Helsinki

To validate our findings, another Finnish patient series from the Helsinki region was analyzed for the respective variants. The additional patients consisted of 121 familial exome- or whole-genome-sequenced breast cancer patients from 77 families. The breast cancer index patients were recruited in the Helsinki University Hospital at the Departments of Oncology in 1997–1998 and 2000 [34,35] and Surgery in 2001–2004 [36], with additional familial patients recruited in the Department of Clinical Genetics [36,37,38]. Altogether, 99 patients were from 56 families with at least three members affected with BC or OC among first- or second-degree relatives; 21 patients had one affected first-degree relative, and 1 breast cancer index patient had a family history of other cancers. In more detail, 17 families also included OC and 9 included male BC; 6 of the breast cancer patients in this study were males. The mean age at breast cancer diagnosis among the patients was 51.4 years (range 28–80 years). No patient had a pathogenic *BRCA1*, *BRCA2*, *TP53, PALB2*, *CHEK2*, *ATM*, *RAD51C*, *RAD51D*, or *FANCM* variant. The genomic DNA used in the exome and genome sequencing was isolated from peripheral blood samples. The study was approved by the Ethics Committee of the Helsinki University Hospital, with informed consent obtained from all patients.

### 2.3. Protein Structure Modeling

The structural change was predicted for the five novel missense variants in the genes *WRN*(R732P), *RNF8*(C55W), *TOP3A*(S395C), *ERCC2*(Q698R), and *TREX2*(R152P). Other three novel variants were not included for protein modeling as these were a splice acceptor variant in *RNF4* and two frameshift variants in *EXO1* and *POLE* genes, respectively. 

The PDB (Protein Data Bank) structures of the proteins were downloaded from the UNIPROT database (WRN-Q14191, RNF8-O76064, TOP3A-Q13472, ERCC2-P18074, TREX2-Q9BQ50). The wild structures were showing a 90% confidence score as per residue in the Alpha Fold Protein Structure Database. The mutations were created into these PDBs using the mutagenesis plugin in PYMOL. These wild and mutated tertiary structures were used for the energy minimization of molecule models with Steepest descent steps 1000, conjugate gradient steps 10, and default amber parameters in UCSF-Chimera [39].

## 3. Results

### 3.1. Turku Whole-Exome Sequencing Set Results

**Figure 1:**
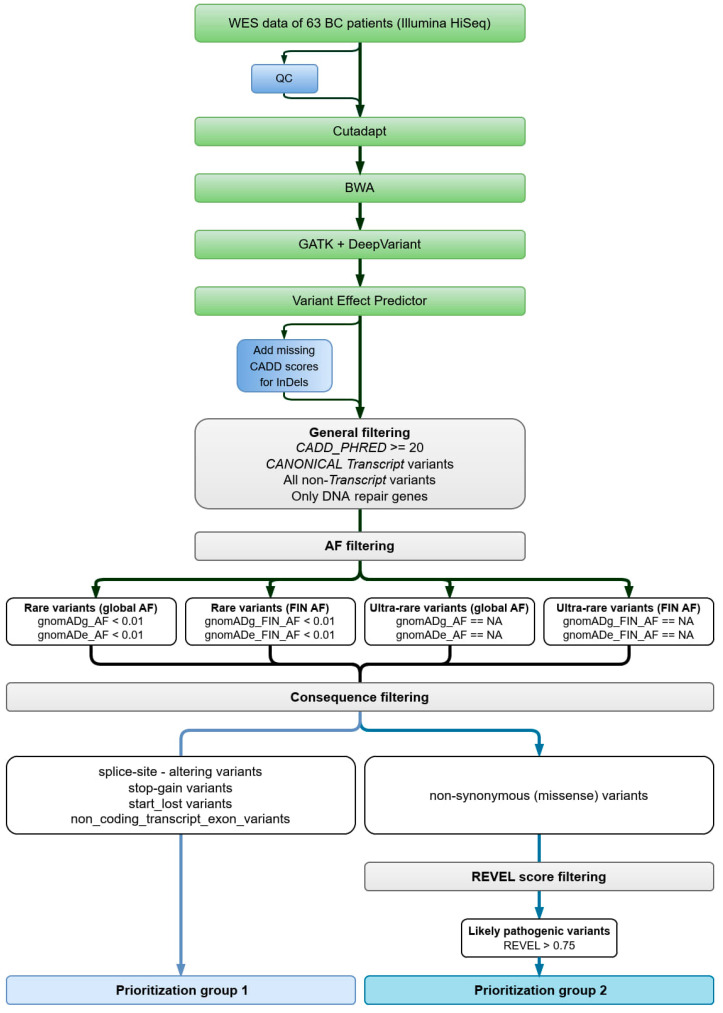
Flow chart describing variant calling and variant prioritization. Variants were called from whole-exome sequencing (WES) data obtained from 63 patients using the Genome Analysis Toolkit (GATK) and DeepVariant software after alignment using the Burrows–Wheeler Alignment Tool (BWA). Consensus variants were annotated using the Ensembl VEP 108. Annotated variants were initially filtered by the Combined Annotation Dependent Depletion (CADD) score and allele frequency (AF) obtained from the Genome Aggregation Database (gnomAD). After the prioritization step using variant consequences, non-synonymous variants were further filtered by REVEL score.

After the analysis pipeline (Figure 1), a total of 72 variants remained for 45 patients (Table 1), while 18 patients had no variants. All detected variants were heterozygous. Patient 433 (marked with ^ in Table 2) with bilateral BC and TNBC diagnosed at the age of 31 years was known to be a homozygote for the intermediate risk variant FANCM c.5101C>T. The patient did not have any other deleterious variants. A total of 27 of our 63 patients (67%) were found to have more than one deleterious variant after all filtering steps. One patient had a maximum of five different deleterious variants (Table 2). 

Eight novel variants were found in eight different genes. Novel variants were *WRN* (chr8:31111721 G/C), *ERCC2* (chr19:45352306 T/C), *TREX2* (chrX:153444976 C/G), *RNF8* (chr6:37360499 C/G), *RNF4* (chr4:2490336 G/T), *EXO1* (chr1:241861448-/T), *POLE* (chr12:132677395-/T), and *TOP3A* (chr17:18292743 T/A). The identified 64 known variants were located in 48 genes (Table 1). Among these, *PALB2* (rs180177100) was the only variant that had been previously linked to BC susceptibility. Each novel variant was identified in one individual only. 

Patient “634” carrying the novel variant in *WRN* (chr8:31111721 G/C) had TNBC (Table 2). The age of onset in this patient was 34. She also had another variant in *WRN* gene (rs11574410) and a novel variant in *POLE* (chr12:13267795-T), as well as a variant in *BRCA1* (rs28897689). This patient did not show a family history of breast cancer, neither did she have Werner syndrome, which is a progeroid syndrome [40] (Table 2). Patient “435” with *RNF8* (chr6:37360499 C/G) variant also did not show a family history of the disease. She was diagnosed at the age of 32. She also carried variants in *WRN* (rs78488552) and *RECQL5* (rs565251228) genes (Table 1 and Table 2). Patient “623” with novel *RNF4* (chr4:2490336 G/T) variant was diagnosed at 31 years of age and had a positive family history of BC. No other variants were found in this study, but she was known to carry a *CHEK2* c.1100delC variant. Patient “440” with novel *ERCC2* (chr19:45352306 T/C) variant had bilateral BC at the age of 34 and 37. She also had variants in *BRCA2* (rs55712212) and *PER1* (rs137923123). 

The most deleterious variant based on its CADD score of 42 was detected in *MLH3* (rs193219754). In our data, this variant was detected in patient “631”. This patient did not have a family history of BC. We also identified one additional deleterious variant in *MLH3* (rs775001669), with a CADD score of 22,6. Patient “72” had this *MLH3* variant together with other variants in *POLQ* (rs41540016) and in *MSH3* (rs199791286). Through our pipeline, we identified five predicted deleterious variants in *POLQ*. One of these variants (rs41540016) was detected in four patients.

### 3.2. Validation Set Helsinki Results

In the validation cohort of 121 patients with familial BC with no existing *BRCA1/2* mutations, 18 of our variants were found and 33 of the patients in the set were found to have at least one of the variants. However, none of the novel variants were detected in this sample set. Five of the patients had multiple variants. Overall, only five patients with a found variant were 40 years or younger at the time of diagnosis. Variants found in these young patients were *PNKP* rs201503405, *BRCA2* rs11571833, *ERCC4* rs1799802, *LIG1* rs3730947, *NEIL1* rs5745908, and *SPIDR* rs187418762. Only two of the CADD>30 variants were found in the validation set. These were *BRCA2* rs11571833 which was found on two patients and *NEIL1* rs5745908 which was found on two patients.

### 3.3. Protein Structure Modeling of Novel Variants

For six exonic novel variants present in this study, we present a lollipop diagram (Supplementary Figure S1), in which the mutation is marked in the amino acid chain with a pin alongside the functional domains. *TREX2* is not shown because of a lack of knowledge of the protein’s functional domains, and *RNF4* is not shown because the detected variant is in a splice acceptor site.

**Figure 2:**
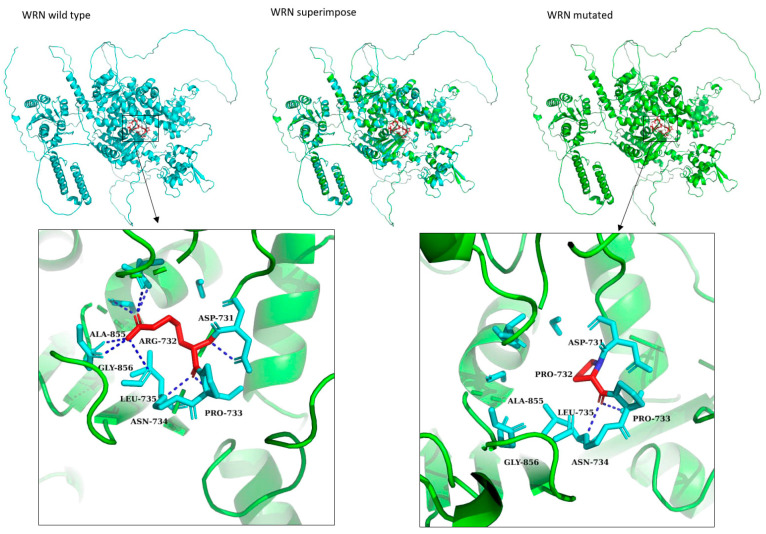
The Werner protein structure. The Werner helicase structure is shown by PYMOL in green color, and the red-color-highlighted variant is arginine, which is changed to proline at 732. (chr8:31111721, G/C) The arginine-732 (red) is bonded (red dotted lines) with Leucine-735, Asparagine-731, and proline-733. Mutation arginine-732-proline as a point mutated structure is shown in red color. Proline-732 is shown bonded with Leucine-735 and Phenylalanine-730. Variant in WRN is Arg732Pro (chr8:31111721, G/C). Superimposition is shown among the wild and mutated structure of Werner helicase by using Chimera software (https://www.cgl.ucsf.edu/chimerax/), accessed on 21 March 2022. The wild-type is shown in cyan color and mutated type is shown in green color.

Novel *WRN* variant was interesting because the amino acid change is located in the DNA binding site of the protein and arginine is replaced by proline. This changes the conformation and bonding in the proteins active site (Figure 2).

For all other novel missense variants in *ERCC2* (chr19:45352306 T/C), *TREX2* (chrX:153444976 C/G), *RNF8* (chr6:37360499 C/G), and *TOP3A* (chr17:18292743 T/A), we present the protein structure model in wild and mutated form. Active sites are presented in wild-type and mutated form in the figure next to the protein structure (Supplementary Figure S2).

## 4. Discussion

This study focused on *BRCA1/2*-negative early-onset BC patients with or without a family history of BC. All identified deleterious variants were classified as damaging by our pipeline. Furthermore, particular alterations were classified as very damaging variants. Due to the strict filtering criteria in our pipeline, a deleterious variant in DNA repair genes was not detected in all patients of the cohort.

### 4.1. Novel Variants Detected

Novel rare variants were identified in genes that have not previously been associated with BC. All novel variants were detected in only a single individual, and none of them were identified in the validation set. Further studies are warranted to elucidate whether these genes can be considered new risk genes for EOBC. It is possible that the respective novel variants are so-called ‘private mutations’ that do not occur in other individuals or families. Thus, additional family members, both affected and healthy at an older age, would need to be analyzed for the segregation of these variants. This knowledge would provide pivotal information to provide accurate genetic counseling for each family.

Although none of the genes, where these novel variants were found in, have been previously regarded as EOBC risk genes, there is limited evidence linking certain variants to cancer development. Especially in the European population, several *WRN* gene variants have been associated with an elevated risk of BC [41,42,43,44]. Interestingly, the same amino acid site of the variant found in our study (p.Arg732Pro) has been demonstrated as a stop codon variant causing Werner syndrome when homozygous [45]. This syndrome is a rare progressive disorder characterized by the appearance of unusually accelerated aging (progeria). It is common for affected Werner syndrome individuals to develop multiple cancers during their lifetime. 

*WRN* is a part of the RecQ family and plays a crucial role in maintaining genomic stability. Importantly, the *WRN* protein interacts directly with *BRCA1* in DNA double-strand breakage (DSB) repair. *BRCA1* binds to the WRN C-terminal area and increases the helicase activity of WRN. Both are essential for maintaining genomic stability in the case of DSB [46]. The novel variant (chr8:31111721 G/C p.Arg732Pro) is located in the middle of the gene between two helicase domains (Figure 2). Based on our modeling, arginine creates multiple bonds, which maintain the stability of the protein. These bonds are lost when arginine is replaced with proline in p.Arg732Pro, causing instability for the structure (Figure 2). Arginine amino acid has a carboxylic (-COOH) group and one basic amino group (-NH2). While the proline amino acid does not have neither a carboxylic group nor an amino group, it comprises five membered nitrogen-containing heterocyclic rings [47].

*RNF8* plays a central role in DNA double-stranded break (DSB) signal transduction. DSB damage is the most toxic type of DNA damage to cells and is related to genomic instability [48]. RNF8 is also an essential factor for the protection of telomere end integrity. Additionally, it takes part in cell cycle regulation [49]. The novel deleterious variant (chr6:37360499 C/G, p.C55W) is situated in the forkhead-associated (FHA) domain (Supplementary Figures S1 and S2), which is a phosphopeptide recognition domain found in many regulatory proteins. The cysteine in RNF8 is a non-essential and polar but uncharged amino acid. Tryptophan is a non-polar essential amino acid in the human body obtained from diet that works as a precursor for neurotransmitter serotonin. Elevated tryptophan levels are reported to be associated with BC [50]. 

One of the novel variants was detected in another RING Finger Protein gene, *RNF4*. The protein encoded by this gene contains a RING finger motif and acts as a transcription regulator. Homology-Directed Repair (HDR) is among its related pathways. To date, there are no studies that show a genetic predisposition to cancer in *RNF4*-associated variants, but this gene has been reported as a somatic mutation in multiple cancers [51]. *RNF4* has been shown to play an independent role in tumor necrosis factor-alpha (TNF-alpha)-mediated cell death. Furthermore, RNF4 has a decisive impact on DNA double-strand break repair [52,53].

The *ERCC2* gene is involved in nucleotide excision repair for the removal of various DNA lesions. Variants in this gene have been studied in Indian, Chinese, and Moroccan populations and have been linked to breast cancer susceptibility [54,55,56]. To the best of our knowledge, this is the first time a deleterious variant in this gene has been identified in Finnish BC patients. The novel *ERCC2* variant was only found in one patient, but we also found three other variants in the same gene.

Via modeling the novel variants with predicted amino acid changes, we conclude that these variants are deleterious. All active sites change dramatically and therefore crucially affect protein function. The dysfunction of these DNA damage repair route proteins will contribute to an elevated mutation load. For the novel variants in *RNF4* and *RNF8*, it is challenging to propose their role in EOBC without any additional data despite their high pathogenicity scores. 

Novel variants were found in combination with other deleterious variants (Table 2), which makes it difficult to identify the likely causative ones. Especially the novel *WRN* variant, since it was only found in combination with another variant in the same gene. This could explain why the novel variants were not seen in the Helsinki cohort validation set, which included BC patients with a family history without a restriction of a particular young age at cancer diagnosis. 

### 4.2. Previously Known Cancer Variants Detected 

All patients in this study had been screened negative for *BRCA1/2* variants. However, we found five variants in these two genes (rs81002862, rs28897758, rs28897689, rs11571833, and rs55712212). Although these variants are considered deleterious in our pipeline, there is prior conflicting evidence suggesting that these are benign [57]. For example, variant rs11571833 (chr13:32398489; c.9976A>T) is a rare truncating mutation in *BRCA2*, associated with low breast cancer risk [58]. Variant rs55712212 (chr13:32341176; c.6821G>T) in *BRCA2* has been classified as likely benign or a variant of unknown significance (VUS) in ClinVar [59]. Therefore, these variants have not been considered pathogenic when study individuals were originally evaluated.

Multiple patients in our study carried more than one predicted deleterious variant (Table 2). Some have previously been associated with other cancer types. The genes *MSH3* and *MLH3* are known cancer genes originally identified in colorectal cancer. Currently, the cancer spectrum associated with these genes has broadened [60]. Importantly for the present study, a later meta-analysis of *MSH3* variant polymorphisms has shown an association of this gene with an increased risk of BC [61,62].

Patient “72” showed variants in both *MSH3* and *MLH3* mismatch DNA repair genes. Hence, these variants may at least be partially responsible for the development of EOBC. Additionally, this patient also had a deleterious variant in *POLQ*. No other cancer cases in the family had been reported. 

*CHEK2* variant rs587782401 has been associated with breast cancer, and it has been classified as likely pathogenic in ClinVar [63]. *CHEK2* is a known breast cancer susceptibility gene that usually causes a moderate risk of breast cancer, and its truncating variants have been associated with a 2- to 3-fold risk of breast cancer [64]. In some families, *CHEK2* variants have been considered to be even more cancer-causing. Many splice site variants in *CHEK2* lead to impaired splicing and very little or no full-length transcripts [65]. *CHEK2* variants have been associated with a higher risk of bilateral breast cancer [66], which was the case with this patient as well.

*FANCI* has been identified as a possible risk gene for BC susceptibility [67]. Loss-of-function variants have been identified in breast cancer patients [68]. *FANCL* variant rs759217526 has also been reported in a Spanish study on familial BC [68]. In ClinVar, it has been reported as both pathogenic and benign [69].

We found a novel *EXO1* variant with a high CADD score. Some *EXO1* variants have been associated with higher susceptibility for breast cancer [70]. A high expression of Exo1 is associated with poor prognosis in BC [71].

In a previous study, it has been noted that *POLQ* is overexpressed in BC, which leads to a poor prognosis, and this overexpression also has effects on key cancer pathways [72]. *POLQ* has been associated with a poor outcome in prostate cancer [73]. In our pipeline, we found four different *POLQ* deleterious variants, of which some variants were also found in the Helsinki familial BC set. 

Some of the known variants, which have previously been associated with other cancer types, were found in the validation cohort of 121 *BRCA1/2* negative familial BC patients. The Helsinki validation set differs from the Turku BC patients, as the Helsinki BC patient cohort comprised patients without a limitation on their age at diagnosis, and all patients had a positive family history, whereas 28.6% of the patients in the original set had no family history of BC. Therefore, our patients present a rather specific subpopulation of EOBC patients. In the original set, the mean age at the time of diagnosis was 33.02 (range 23–40 years), and in Helsinki set the mean was 51.4 years (range 28–80 years). All Turku EOBC patients had an onset of cancer before or at the age of 40 years. 

### 4.3. Polygenic Variants Detected

In this study, several patients had more than a single deleterious variant (Table 2), and 28.6% did not fulfill the Lund criteria. Only a few patients carried several deleterious variants in the Helsinki validation set. An earlier study by Määttä et al. described a different patient group and only included few patients with multiple PVs [10]. The previous study focused on families with several BCs in direct lineage without limitations with respect to the age at diagnosis. Based on these studies, we suggest that multiple deleterious or pathogenic variants are characteristic for EOBC regardless of family history. Our findings suggest that there are distinct BC subgroups with different genetic backgrounds. This is also in line with the fact that PVs have rarely been recognized in EOBC patients up to date. Our results demonstrate that further studies are warranted to identify optimal genetic testing in different BC subgroups. Moreover, this study sheds new light on the yet unrecognized EOBC subgroup profiles.

Breast cancer risk factors such as personal and family history, breast histopathology, lifestyle factors, high- and moderate-risk gene PVs, polygenic risk score (PRS), and prediction models such as the Breast and Ovarian Analysis of Disease Incidence and Carrier Estimation Algorithm (BOADICEA) can be used to stratify the individual risk of breast cancer [74]. We found that a considerable fraction of the analyzed young breast cancer patients tended to carry multiple deleterious variants in DNA repair genes or even multiple variants in the same gene. The results of our study support the importance of a polygenic model in risk stratification. 

### 4.4. The Homogenic Group of the Study’s EOBC Patients

The strength of this study is our well-selected, early-onset patient group without known pathogenic gene variations in *BRCA1/2* genes. In this study, the patient cohort was well characterized and derived from a homogenous population (the Finnish population is a well-known founder population due to its strong genetic isolation over centuries). Our analysis was designed to detect rare, highly deleterious, and possibly pathogenic variants. For example, a well-known *CHEK2* variant 1100delC that is known to moderately increase the risk of young-onset BC [38] was not detectable in our pipeline.

### 4.5. Limitations of the Study

Despite the very promising results, there are still many steps to be taken before these results can be used for clinical counseling. Functional analysis of the novel variants is warranted, as well as a validation of these findings in larger and more diverse cohorts. Our study focused purely on the DNA repair pathway genes, which exclude all deleterious variants in other genes. These variants can later be addressed, as WES was conducted.

## 5. Conclusions

This study identified multiple potential deleterious variants in DNA repair pathway genes. Novel deleterious variants were observed in the genes *WRN, ERCC2, TREX2, RNF8, RNF4, EXO1, POLE*, and *TOP3A*, which may partially explain EOBC. Furthermore, protein structure modeling supports the conclusion that the novel variants identified could potentially be pathogenic. Many of the identified variants have been previously associated with other cancer types. Additionally, we found an unexpectedly high number of patients carrying multiple variants. This presents multiple interesting cases at an individual level, as well as on a larger scale. Our findings suggest that EOBC patients without a family history should also be screened for pathogenic variants in a multigene panel, similar to the current routine treatment for outpatients. However, additional analyses are warranted, and our results require validation in larger cohorts.

Novel knowledge about polygenic risk factors may contribute to improvements in personalized screening and treatment modalities [75]. Our study sheds crucial light on the genetic architecture of EOBC and emphasizes that the polygenic model plays a pivotal role in unraveling its etiology. 

## Figures and Tables

**Table 1:** Found variants. First column contains rs-number or bolded chromosomal position with the nucleotide change if no rs-number was available. Location: GRCh38. Consequence: the row is marked gray if variants’ consequences are stop gain, start lost, splice acceptor, or splice donor; these belong to Group 1. White rows are non-synonymous variants, which belong to Group 2. Group 2 is divided into two subcategories, of which subcategory 1 meets the criteria of AF < 0.01 and REVEL > 0.75 or missing. Subcategory 2 meets the criteria of AF < 0.02 and REVEL > 0.4. SIFT score: deleterious (0–0.05), tolerated (0.06–1). PolyPhen score: probably damaging (0.85–1.0), possibly damaging (0.15–0.85), benign (0–0.15). REVEL: range 0–1; higher score signals greater likelihood for variant to be disease-causing. CADD-Phred: higher score predicts the variant to be more deleterious, e.g., score >20 signals it belonging to 1% most deleterious, >30 to 0.1% most deleterious, and >40 to 0.01%.

Variant	Gene	Location	Nucleotide Change	Effect on Protein	Consequence	Subcategory	gnomADe FIN AF	gnomADg FIN AF	SIFT	PolyPhen	REVEL	CADD PHRED
rs193219754	*MLH3*	chr14:75039918	c.3563C>G	p.Ser1188Ter	stop gained		0.002491	0.003159	NA	NA	NA	42
rs180177100	*PALB2*	chr16:23635306	c.1240C>T	p.Arg414Ter	stop gained		0	0	NA	NA	NA	37
rs11574410	*WRN*	chr8:31173019	c.4216C>T	p.Arg1406Ter	stop gained		0.0006469	0.0002832	NA	NA	NA	36
rs81002862	*BRCA2*	chr13:32380005	c.9118-2A>G	NA	splice acceptor		0.0003697	0.0001885	NA	NA	NA	35
rs11571833	*BRCA2*	chr13:32398489	c.9976A>T	p.Lys3326Ter	stop gained		0.01086	0.01045	NA	NA	NA	35
rs5745908	*NEIL1*	chr15:75349341	c.434+2T>C	NA	splice donor		0.0018	0.001508	NA	NA	NA	34
rs587782401	*CHEK2*	chr22:28734401	c.319+2T>A	NA	splice donor		0.0005548	0.0003764	NA	NA	NA	34
rs766240074	*RAD54L*	chr1:46273748	c.1610+1G>A	NA	splice donor		4.968 × 10^−5^	0	NA	NA	NA	33
**chr4:2490336 G/T**	***RNF4***	chr4:2490336	c.-157-1G>T	NA	splice acceptor		NA	NA	NA	NA	NA	33
rs756188698	*RAD18*	chr3:8899048	c.1169-1G>C	NA	splice acceptor		0.0002398	9.457 × 10^−5^	NA	NA	NA	28.9
rs11572913	*GTF2H3*	chr12:123633862	c.3G>A	p.Met1?	start lost		0.0007112	0.000565	0.02lc	0.273	NA	24.8
rs187418762	*SPIDR*	chr8:47713484	c.2189-5G>A	NA	splice region, splice polypyrimidine tract, intron		0.01286	0.01198	NA	NA	NA	20.5
rs149243307	*FANCI*	chr15:89260841	c.286G>A	p.Glu96Lys	missense, splice region	2	0.001848	0.001507	0	1	0.448	33
rs41540016	*POLQ*	chr3:121436275	c.7390G>A	p.Ala2464Thr	missense, splice region	2	0.01381	0.01208	0	1	0.84	33
**chr1:241861448 -/T**	***EXO1***	chr1:241861447-241861448	c.987dup	p.Lys330Ter	frameshift	1	NA	NA	NA	NA	NA	33
rs773570504	*ATM*	chr11:108326152-108326153	c.6908dup	p.Glu2304GlyfsTer69	frameshift	1	4.625 × 10^−5^	9.56 × 10^−5^	NA	NA	NA	33
rs762390984	*FANCI*	chr15:89301393-89301405	c.2957_2969del	p.Val986AlafsTer39	frameshift	1	0.002957	0.002454	NA	NA	NA	33
rs759217526	*FANCL*	chr2:58159793-58159794	c.1096_1099dup	p.Thr367AsnfsTer13	frameshift	1	0.001814	0.002649	NA	NA	NA	33
rs199791286	*MSH3*	chr5:80778737	c.2336G>A	p.Arg779His	missense	1	0.001294	0.001415	0	1	0.884	32
**chr8:31111721 G/C**	***WRN***	chr8:31111721	c.2195G>C	p.Arg732Pro	missense	2	NA	NA	0	1	0.499	31
**chr12:132677395 -/T**	***POLE***	chr12:132677394-132677395	c.769dup	p.Ile257AsnfsTer6	frameshift	1	NA	NA	NA	NA	NA	31
rs28363284	*RAD51D*	chr17:35103294	c.698A>G	p.Glu233Gly	missense	1	0.001185	0.001797	0.04	0.973	NA	29.9
rs746243211	*MSH3*	chr5:80672754	c.923A>T	p.Lys308Met	missense	1	0	0	0	1	0.823	29.7
rs137923123	*PER1*	chr17:8147713	c.1349G>A	p.Arg450His	missense	1	0.003681	0.002823	0	1	NA	29.2
rs373080718	*TOP3A*	chr17:18277728	c.2774C>T	p.Pro925Leu	missense	1	4.623 × 10^−5^	0	0.01	0.999	NA	29
rs2306211	*POLQ*	chr3:121432937	c.7640C>T	p.Ala2547Val	missense	2	0.007347	0.006223	0.03	1	0.571	28.8
rs200535477	*RECQL5*	chr17:75627670	c.2828G>A	p.Arg943His	missense	1	0.009798	0.008201	0	1	NA	28.7
rs202068855	*RPA1*	chr17:1880615	c.1165C>T	p.Arg389Trp	missense	2	0.01502	0.0152	0	1	NA	28.1
rs144564120	*ERCC2*	chr19:45352249	c.2150C>G	p.Ala717Gly	missense	1	4.624 × 10^−5^	0	0.01	0.015	NA	28.1
rs28897758	*BRCA2*	chr13:32394734	c.1267T>G	p.Cys423Gly	missense	1	0.0004619	0.000659	0	1	0.788	28
rs34001746	*TOP3A*	chr17:18285268	c.1751T>G	p.Leu584Arg	missense	1	0.0004619	0.000659	0.02	0.803	NA	27.3
rs121913016	*ERCC2*	chr19:45357368	c.1381C>G	p.Leu461Val	missense	1	4.633e-05	0	0	0.982	NA	27.3
rs1805378	*NTHL1*	chr16:2044652	c.503T>C	p.Ile168Thr	missense	1	0.0004352	0.0003766	0	1	0.876	27.1
rs34642881	*RECQL4*	chr8:144517415	c.212A>G	p.Glu71Gly	missense, splice region	1	0.004511	0.005941	0.02	0.803	NA	26.6
rs17879961	*CHEK2*	chr22:28725099	c.470T>C	p.Ile157Thr	missense	1	0.004511	0.005941	0.07	0.514	NA	26.5
rs142213781	*NEIL1*	chr15:75353853	c.833C>T	p.Thr278Ile	missense	2	0.002086	0.002352	0	1	0.563	26.4
rs562132292	*ERCC2*	chr19:45357290	c.1459C>T	p.Arg487Trp	missense	1	0.0003272	0.0006591	0	0.998	NA	26.4
**chr6:37360499 C/G**	***RNF8***	chr6:37360499	c.165C>G	p.Cys55Trp	missense	2	NA	NA	0	1	0.747	26.3
rs149253459	*FAAP100*	chr17:81547649	c.1433A>G	p.Gln478Arg	missense	1	0.002894	0.0016	0.01	0.997	NA	26.2
rs750771205	*ATM*	chr11:108289000	c.4133C>T	p.Pro1378Leu	missense	2	0	0	0	0.969	0.546	26.1
rs1801673	*ATM*	chr11:108304736	c.5558A>T	p.Asp1853Val	missense	2	0.003699	0.00292	0	0.987	0.589	26.1
rs1799802	*ERCC4*	chr16:13934224	c.1135C>T	p.Pro379Ser	missense	2	0.007207	0.009714	0	1	0.526	25.4
rs11212587	*ATM*	chr11:108315883	c.6067G>A	p.Gly2023Arg	missense	2	0.0008318	0.0007553	0	0.364	0.511	25.3
rs61752784	*POLG*	chr15:89330133	c.803G>C	p.Gly268Ala	missense	1	0.004158	0.00546	0	0.999	0.967	25.3
rs200981995	*LIG3*	chr17:34999827	c.2302T>C	p.Tyr768His	missense	2	0.01173	0.009134	0.11	0.994	NA	25.1
**chr17:18292743 T/A**	***TOP3A***	chr17:18292743	c.1183A>T	p.Ser395Cys	missense	1	NA	NA	0.01	0.976	NA	25
rs201920810	*SPIDR*	chr8:47712713	c.2029G>A	p.Asp677Asn	missense	2	0	0	0	1	0.561	24.9
rs140566004	*POLE*	chr12:132673646	c.1288G>A	p.Ala430Thr	missense	2	0.0001395	0	0	0.997	0.487	24.7
rs78488552	*WRN*	chr8:31154721	c.3785C>G	p.Thr1262Arg	missense	2	0.000231	9.432 × 10^−5^	0	0.999	0.413	24.5
rs546221341	*POLQ*	chr3:121488663-121488669	c.4262_4268del	p.Ile1421ArgfsTer8	frameshift	1	0.006209	0.005384	NA	NA	NA	24.3
rs145289229	*POLG*	chr15:89328532	c.1174C>G	p.Leu392Val	missense	1	0.007823	0.01085	0.06	0.999	0.796	24.2
rs55748151	*POLQ*	chr3:121533021	c.929T>G	p.Val310Gly	missense	2	0.001063	0.001413	0	0.552	0.498	24.1
rs150018949	*EXO5*	chr1:40515573-40515574	c.1029_1030insG	p.Arg344AlafsTer10	frameshift	1	0.004352	0.003488	NA	NA	NA	24.1
rs771308001	*ERCC1*	chr19:45407145-45407146	NA	NA	downstream gene	1	0.003376	0.00311	NA	NA	NA	23.7
rs565251228	*RECQL5*	chr17:75624891	NA	NA	downstream gene	1	0.0001461	0.0002827	NA	NA	NA	23.4
rs55801750	*ATM*	chr11:108330296	c.7390T>C	p.Cys2464Arg	missense	2	4.619 × 10^−5^	0	0.1	0.005	0.668	22.9
rs201503405	*PNKP*	chr19:49862573	c.901C>T	p.Arg301Trp	missense	1	0.006464	0.006775	0	0.911	NA	22.9
**chr19:45352306 T/C**	***ERCC2***	chr19:45352306	c.2093A>G	p.Gln698Arg	missense	1	NA	NA	0.54	0.218	NA	22.8
**chrX:153444976 C/G**	***TREX2***	chrX:153444976	c.455G>C	p.Arg152Pro	missense	1	NA	NA	0	0.999	NA	22.7
rs775001669	*MLH3*	chr14:75048767	c.889C>T	p.Arg297Trp	missense	2	0.00134	0.001132	0	1	0.655	22.6
rs41549716	*POLG*	chr15:89321842	c.2492A>G	p.Tyr831Cys	missense	2	0.01803	0.0161	0.02	0.995	0.732	22.6
rs201414369	*EME1*	chr17:50380824	c.1598G>A	p.Arg533His	missense	1	0.0001386	0.0001884	0.22	0.994	NA	22.6
rs144340710	*TP53*	chr17:7674259	c.704A>G	p.Asn235Ser	missense	1	0.0002772	0.0004745	0.22	0.385	NA	22.5
rs4987202	*RAD23A*	chr19:12948812	c.599C>T	p.Thr200Met	missense, splice region	1	0.004359	0.003299	0.43	0.02	NA	22.5
rs146309259	*LIG1*	chr19:48121298	c.2257G>A	p.Val753Met	missense	1	0.002451	0.001788	0.06	0.407	NA	22.5
rs28897689	*BRCA1*	chr17:43091492	c.4039A>G	p.Arg1347Gly	missense	1	0.001944	0.001788	0.09	0.255	NA	22.2
rs55712212	*BRCA2*	chr13:32341176	c.6821G>T	p.Gly2274Val	missense	2	0.01303	0.01349	0.37	0.966	0.481	21.8
rs144276604	*XAB2*	chr19:7625912	c.790G>A	p.Asp264Asn	missense	1	0.0002318	0	0.09	0.846	NA	21.7
rs3730947	*LIG1*	chr19:48140013	c.1045G>A	p.Val349Met	missense	2	0.01564	0.01648	0	0.997	NA	21.5
rs776329282	*ERCC4*	chr16:13926750-13926755	c.580_584+1del	NA	in-frame deletion, splice region	1	9.256 × 10^−5^	0.0001884	NA	NA	NA	20.5
rs763165669	*NTHL1*	chr16:2038404-2038416	NA	NA	downstream gene	2	0.01363	0.007775	NA	NA	NA	NA
rs41547220	*POLQ*	chr3:121489857-121489859	c.3072_3074del	p.Lys1025del	in-frame deletion	2	0.01831	0.01925	NA	NA	NA	NA

**Table 2:** Clinical data of the EOBC patients. ID column: patient identification number. The ‘–’ after ID number means negative Lund criteria. Found var columns include the variants’ rs-numbers and genes. Novel variants are bolded. Age is the age of the patient at breast cancer diagnosis. If two ages are shown, the patient had bilateral cancer. Last column includes histology, grade, and hormonal markers. Triple-negative cancers are bolded. Bilateral breast cancer is marked with 2, after which the same information is presented for the second cancer. Information was not available for all patients from patient files. If a part is left blank, no information was available. Gray marked variants’ consequences are stop gain, start lost, splice acceptor, or splice donor; these belong to Group 1. Novel variants and TNBC are bolded.

ID*	Found var					Age	Histology, Grade, ER, PR, Her2
424	rs145289229	rs11571833	rs2306211	rs546221341		25	Ductal, G3, ER+, PR-, Her2+
	*POLG*	*BRCA2*	*POLQ*	*POLQ*			
425						27	Ductal, G3, ER+, PR+, Her2-
						
426						27	Ductal, G3, ER-, PR-, NA
						
427						28	Ductal, G3, ER+, PR+, Her2+
						
428	rs11571833	rs3730947				28	**Ductal, G3, ER-, PR-, Her2-**
	*BRCA2*	*LIG1*					
429						28	Ductal, G3, ER+, PR+, Her2+
						
431	rs140566004	rs4987202				28	Ductal, G3, ER-, PR+, Her2+
	*POLE*	*RAD23A*					
432-	rs201920810	rs773570504				30	Ductal, G3, ER+, PR+, Her2-
	*SPIDR*	*ATM*					
433						31, 35	**Ductal, G3, ER-, PR-, Her2-;** 2 Ductal, ER+, Her2- ^
						
434-	rs200535477	rs2306211				31	**Ductal, G3, ER-, PR-, Her2-**
	*RECQL5*	*POLQ*					
435-	***Novel RNF8***	rs78488552	rs565251228			32	Ductal, G3, ER-, PR-, Her2+
		*WRN*	*RECQL5*				
436-	rs1801673	rs762390984	rs763165669			32	Micropapillar, ER+, PR+, Her2-
	*ATM*	*FANCI*	*NTHL1*				
437	rs373080718	rs202068855				33	DCIS, G3
	*TOP3A*	*RPA1*					
438-	rs187418762					33	**Ductal, G3, ER-, PR-, Her2-**
	*SPIDR*						
440	rs55712212	rs137923123	***novel ERCC2***			34, 37	Ductal, G3, ER-, PR-; 2 LCIS
	*BRCA2*	*PER1*					
441-						34	Ductal, G3, ER+, PR+, Her2-
						
442	rs41547220	rs149253459				34	Ductal, G3, ER+, PR+, Her2-
	*POLQ*	*FAAP100*					
443-	rs766240074	rs562132292				34	**Ductal, G3, ER-, PR-, Her2-**
	*RAD54L*	*ERCC2*					
444-						35	Ductal, G3, ER-, PR-, Her2+
						
445	rs587782401					35,43	Ductal, G3, ER-, PR-, Her2+; 2 Ductal, G3, ER-, PR-, Her2+
	*CHEK2*						
446-	rs771308001					34	Ductal, G2, ER+, PR+, Her2-
	*ERCC1*						
610	rs28897758	rs1805378				40, 40	Ductal, G1, ER+, PR+, Her2-; 2 G3, ER+, PR+, Her2-
	*BRCA2*	*NTHL1*					
611						40, 54	Ductal, G2, ER+, PR-, Her2-; 2 ductal, G2, ER+, PR+, Her2-
						
612	rs81002862	rs11212587				40	DCIS
	*BRCA2*	*ATM*					
613						35	
						
614						26	DCIS, G3
						
615	rs55748151					30	**Ductal, G3, ER-, PR-, Her2-**
	*POLQ*						
616	rs55801750	rs34001746	rs144276604			38	Ductal, G3, ER-, PR-, Her2+
	*ATM*	*TOP3A*	*XAB2*				
617	rs746243211	***Novel Trex2***				40, 67	2 Ductal G3, ER+, PR+, Her2-
	*MSH3*						
618	rs776329282					27	Ductal, G2, ER+, PR+, Her2-
	*ERCC4*						
619	rs5745908	rs41549716	***Novel Top3A***	rs41540016		27	**Ductal, G3, ER-, PR-, Her2-**
	*NEIL1*	*POLG*		*POLQ*			
61	rs121913016	rs180177100	rs144564120			36	Ductal, ER+, PR+, Her2-
	*ERCC2*	*PALB2*	*ERCC2*				
620						35	Ductal, G2, ER+, PR+, Her2-
						
621	rs55712212					39	Lobular
	*BRCA2*						
622						38	Ductal, G2, ER+, PR+, Her2+
						
623	***Novel RNF4***					31	Ductal, G2, ER+, PR+, Her2+
						
624	rs149243307	rs61752784	rs17879961	***Novel EXO1***		23	Ductal, G3, ER+, PR+, Her2+
	*FANCI*	*POLG*	*CHEK2*				
625	rs3730947					28	Lobular, G2, ER+, PR+, Her2-
	*LIG1*						
626	rs750771205	rs145289229	rs146309259	rs3730947	rs587782401	34, 34	Ductal, ER+, PR+, Her2+; 2 DCIS
	*ATM*	*POLG*	*LIG1*	*LIG1*	*CHEK2*		
627						33	
						
628	rs28363284	rs41540016				28	**Ductal, G3, ER-, PR-, Her2-**
	*RAD51D*	*POLQ*					
629-	rs150018949					40	
	*EXO5*						
62						36	DCIS
						
630	rs1799802					26	**Ductal, G3, ER-, PR-, Her2-**
	*ERCC4*						
631-	rs193219754					31	Ductal, G2, ER+, PR-, Her2-
	*MLH3*						
632	rs187418762	rs4987202	rs201503405			38, 67	Ductal; 2 ductal, G3, ER+, PR+, Her2-
	*SPIDR*	*RAD23A*	*PNKP*				
633-	rs41540016					32	Lobular, G2, ER+, PR+, Her2-
	*POLQ*						
634-	***Novel POLE***	rs28897689	***Novel WRN***	rs11574410		34	**Ductal, G3, ER-, PR-, Her2-**
		*BRCA1*		*WRN*			
635-	rs144340710	rs3730947				30	Ductal, G3, ER+, PR-, Her2-
	*TP53*	*LIG1*					
636	rs4987202					24	
	*RAD23A*						
637	rs150018949					31	Ductal, G3, ER+, PR+, Her2+
	*EXO5*						
639-	rs759217526					24	Ductal, G3, ER+, PR+, Her2-
	*FANCL*						
63	rs11572913					37, 46	Ductal, G2; 2 Ductal, G2, ER+, PR+, Her2-
	*GTF2H3*						
64						37, 57	Ductal, G3, ER+, PR+, Her2-; 2
						
65	rs34642881					38	
	*RECQL4*						
66	rs202068855	rs756188698				39	Ductal, G3, ER+, PR+, Her2-
	*RPA1*	*RAD18*					
67	rs142213781					39	Ductal, G1, ER+, PR+, Her2-
	*NEIL1*						
68						39, 47	Lobular, G2, ER+, PR+, Her2-; 2 DCIS, G3
						
69	rs200981995	rs17879961	rs201503405			40	**Ductal, G3, ER-, PR-,Her2-**
	*LIG3*	*CHEK2*	*PNKP*				
71						29	**Ductal, G3, ER-, PR-, Her2-**
						
72-	rs775001669	rs41540016	rs199791286			39	Ductal, G3, ER+, PR+, Her2+
	*MLH3*	*POLQ*	*MSH3*				
73-						33	DCIS, G3
						
74-	rs201414369	rs150018949				36, 36	**Ductal, G3, ER-, PR-, Her2-; 2 ductal, G3, ER-, PR-, Her2-**
	*EME1*	*EXO5*					

## Data Availability

Sequencing data cannot be shared publicly because the data consist of sensitive patient data. More specifically, the data consist of individual clinical data and individual genotypes for young adults. Data are available from the researchers/Turku University Hospital’s Ethics Committee for researchers who meet the criteria for access to confidential data. All other data generated or analyzed during this study are included in this published article or available from the corresponding author upon a reasonable request.

## Supplementary Materials

The following supporting information can be downloaded at: https://www.mdpi.com/article/10.3390/cancers16172955/s1, **Supplementary Figure S1:** Lollipop diagrams showing the position of the novel variants in the gene. The pin shape (the “lollipop”) indicates the position of the identified novel variant in the gene. The position of the variants indicates that they play a key role in the formation of the protein. **Supplementary Figure S2**: Protein structures. Protein structures are shown by PYMOL in column A. Mutation site is marked with a square, and wild-type amino acid is highlighted with different color. In column B, each protein’s wild-type amino acid (highlighted color) is shown with bonding (dotted lines) to other amino acids. In column C, a mutated site with a replacing amino acid (highlighted color) is shown with bonding (dotted lines) to other amino acids.

## Abbreviations

BC	Breast cancer
DCIS	Ductal carcinoma in situ
DSB	Double-strand break
EOBC	Early-onset breast cancer
ER	Estrogen receptor
HER2	Human epidermal growth factor receptor 2
LCIS	Lobular carcinoma in situ
PR	Progesterone receptor
PRS	Polygenic risk score
PV	pathogenic variant
TNBC	Triple-negative breast cancer
VUS	Variant of unknown significance
