# Supplementary material for: Rare Germline Variants in DNA Repair Genes Detected in BRCA-Negative Finnish Patients with Early-Onset Breast Cancer
Source: Cancers (Basel). 2024 Aug 24;16(17):2955. doi: 10.3390/cancers16172955 (PMC11393874; doi:10.3390/cancers16172955)
Supplement: Supplementary file 1 [file cancers-16-02955-s001.zip › cancers-3097819-supplementary/cancers-3097819-Supplementary.pdf]

Supplementary figure 1: Lollipop diagrams showing the position of the novel variants in the gene. The pin shape (the “lollipop”) indicates the position of the identified novel variant in the gene. The position of the variants indicate that they play a key role in the formation of the protein.

Novel *WRN* variant was very interesting because the amino acid change is located in the DNA binding site of the protein and Arginine is replaced Proline. This changes the conformation and bonding in the proteins active site (figure 2).

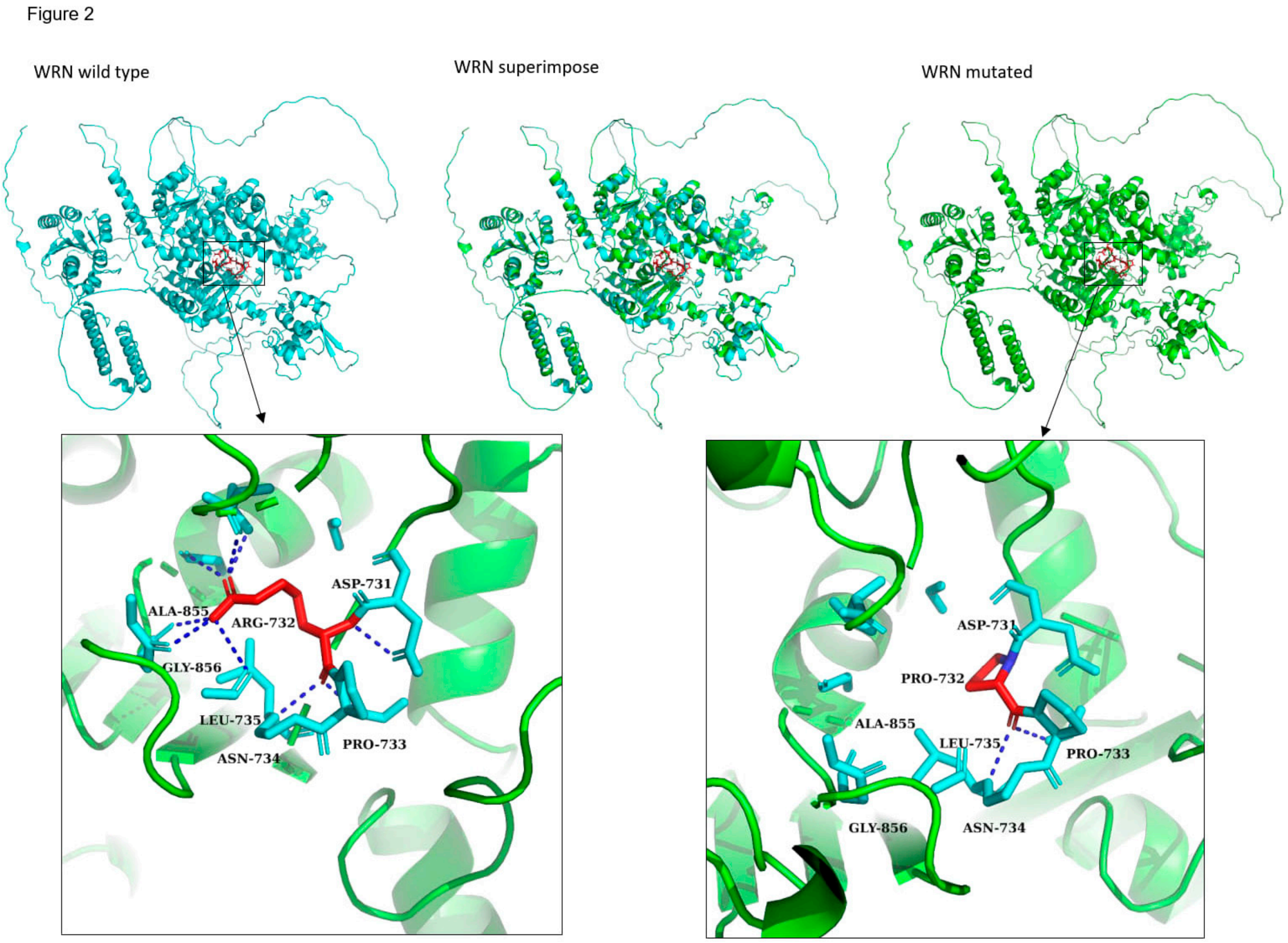

Figure 2: **The Werner protein structure**. A. The Werner helicase structure is shown by PYMOL in green color and red color highlighted variant is Arginine, which is changed to Proline at 732. (chr8:31111721, G/C) B. The Arginine-732 (red) is bonded (red dotted lines) with Leucine-735, Asparagine-731, and Proline-733 C. Mutation Arginine-732-Proline as a point mutated structure is shown in red color. Proline-732 is shown bonded with Leicine-735 and Phenylalanine-730. Variant in WRN is Arg732Pro

(chr8:31111721, G/C). Superimposition is shown among wild and mutated structure of Werner helicase by using Chimera software. The wild type is shown in cyan color and mutated type is shown in green color.

For all other novel missense variants in *ERCC2* (chr19:45352306 T/C), *TREX2* (chrX:153444976 C/G), *RNF8* (chr6:37360499 C/G) and *TOP3A* (chr17:18292743 T/A) we present the protein structure model in wild and mutated form. Active sites are presented in wild type and mutated form in the figure next to the protein structure (Supplementary figure 2).

Supplementary figure 2: **Protein structures.** Protein structures shown by PYMOL in column A. Mutation site is marked with square and wild type amino acid is highlighted with different color. In column B, each protein's wild type amino acid (highlighted color) is shown with bonding (dotted lines) to other amino acids. In column C mutated site with replacing amino acid (highlighted color) is shown with bonding (dotted lines) to other amino acids.
