# Supplementary figures and images for: Rare Germline Variants in DNA Repair Genes Detected in BRCA-Negative Finnish Patients with Early-Onset Breast Cancer
Source: Cancers (Basel). 2024 Aug 24;16(17):2955. doi: 10.3390/cancers16172955 (PMC11393874; doi:10.3390/cancers16172955)

Supplementary Figure 1

A

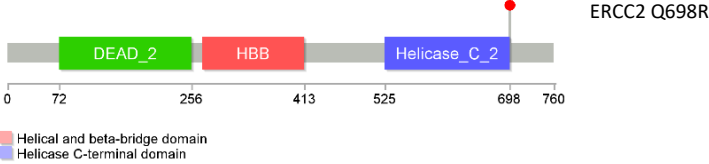

B

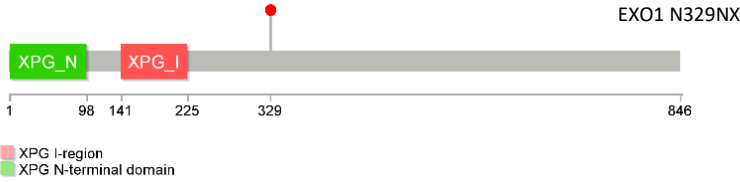

C

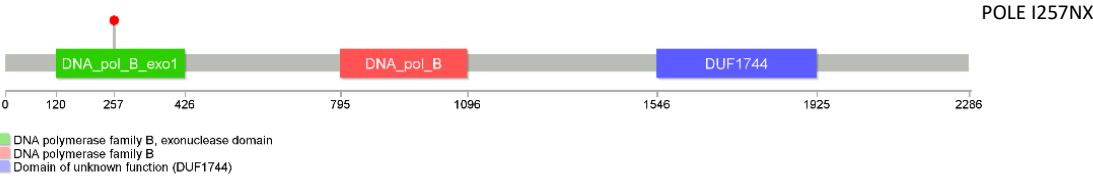

D

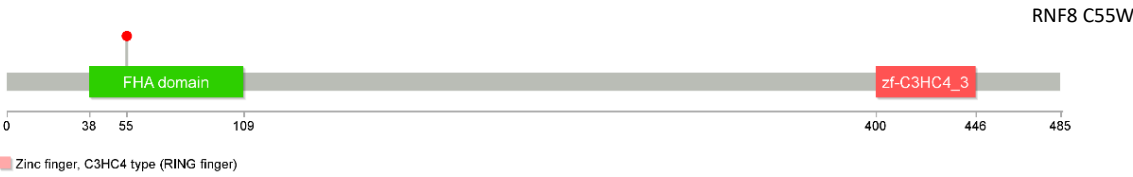

E

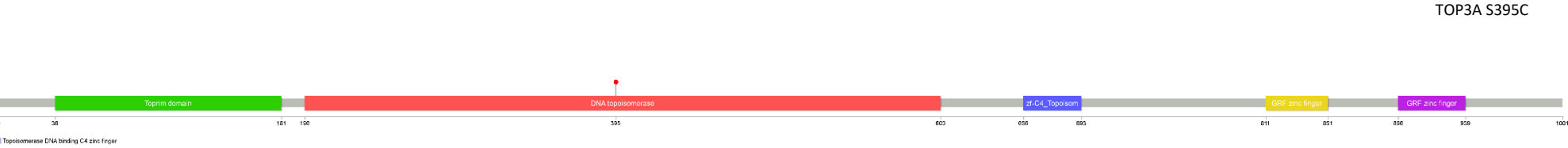

F

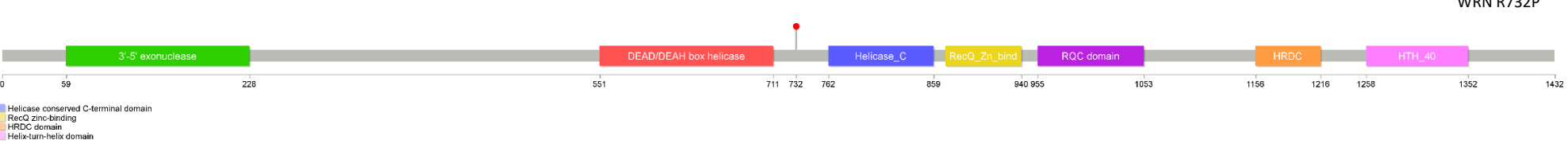

Supplement: Supplementary file 1 [file cancers-16-02955-s001.zip › cancers-3097819-supplementary/supplementary figure 1, lollipop 6.3.2024.pdf]

Supplementary Figure 2

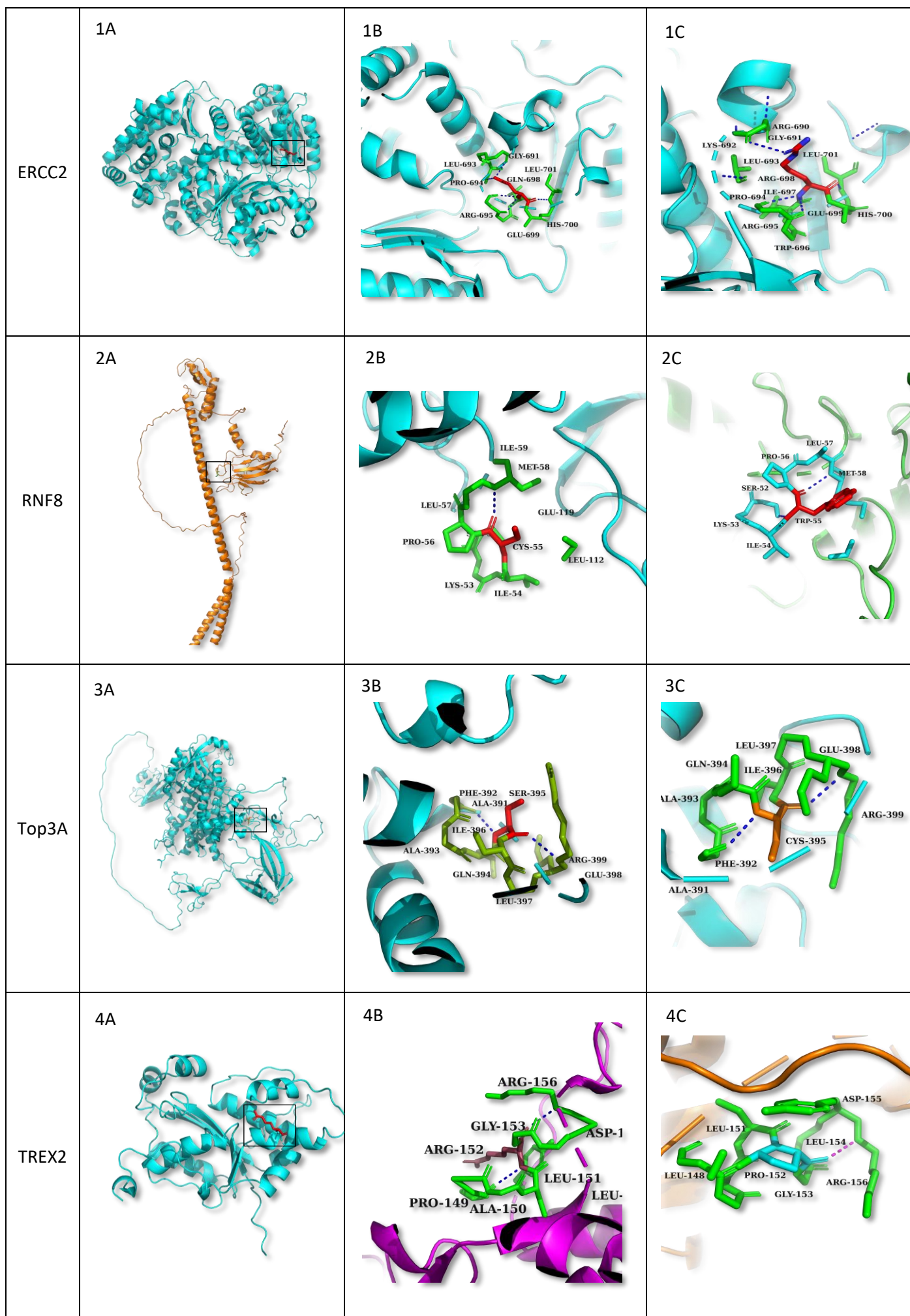

Supplement: Supplementary file 1 [file cancers-16-02955-s001.zip › cancers-3097819-supplementary/supplementary figure 2, Protein structure panel 1.3.2024.pdf]
